# Objective identification and analysis of physiological and behavioral signs of schizophrenia

**DOI:** 10.3109/09638237.2015.1019048

**Published:** 2015-07-20

**Authors:** Maxim Osipov, Yashar Behzadi, John M. Kane, Georgios Petrides, Gari D. Clifford

**Affiliations:** ^a^ Department of Engineering Science, Institute of Biomedical Engineering, University of Oxford, Oxford, UK; ^b^ Proteus Digital Health, Inc., Redwood City, CA, USA; ^c^ Hofstra North Shore-LIJ School of Medicine, Hofstra University, Hempstead, NY, USA

**Keywords:** Activity, heart rate, multiscale entropy, schizophrenia, transfer entropy

## Abstract

*Background*: A patient’s physical activity is often used by psychiatrists to contribute to the diagnostic process for mental disorders. Typically, it is based mostly on self-reports or observations, and hardly ever upon actigraphy. Other signals related to physiology are rarely used, despite the fact that the autonomic nervous system is often affected by mental disorders.

*Aim*: This study attempted to fuse physiological and physical activity data and discover features that are predictive for schizophrenia.

*Method*: Continuous simultaneous heart rate (HR) and physical activity recordings were made on 16 individuals with schizophrenia and 19 healthy controls. Statistical characteristics of the recorded data were analyzed, as well as non-linear rest–activity measures and disorganization measures.

*Results*: Four most predictive features for schizophrenia were identified, namely, the standard deviation and mode of locomotor activity, dynamics of Multiscale Entropy change over scales of HR signal and the mean HR. A classifier trained on these features provided a cross-validation accuracy of 95.3% (AUC = 0.99) for differentiating between schizophrenia patients and controls, compared to 78.5 and 85.5% accuracy (AUC = 0.85 and AUC = 0.90) using only the HR or locomotor activity features.

*Conclusion*: Physiological and physical activity signals provide complimentary information for assessment of mental health.

## Introduction

Schizophrenia is a chronic illness, affecting about 1% of the population. It is characterized by delusions, hallucinations, disorganization of speech and behavior. Onset of schizophrenia usually occurs in early adult years and during the course of the disorder symptoms may wax and wane, or remain relatively stable. Common complications include depression and suicide (about 50% of patients attempt and approximately 10% succeed), usually early in the course of the illness (American Psychiatric Association, 2000). The broad range and diverse combinations of symptoms make differential diagnosis of schizophrenia a complex task that requires a good knowledge of patient’s history. In addition, the early detection of psychotic relapse/exacerbation often depends on patient self-report which is a particular challenge in an illness in which insight is frequently impaired. Psychotic relapse is a continual threat, largely because of high rates of non-adherence in medication taking. Objective measures which could alert clinicians and caregivers to early signs of relapse would have enormous public health significance.

The study presented in this article follows the approach proposed by Lacey in 1959 (Lacey, 1959), who envisioned the use of physiological factors in psychotherapeutic monitoring. This work aimed to extract important features of schizophrenia from measurements of peripherally accessible functions such as heart rate (HR) and locomotor activity. These features were further evaluated for their diagnostic accuracy by identifying the optimal combination of features using a machine learning framework.

## Background

Symptoms related to physical activity play an important role in the diagnosis and clinical monitoring of many patients (American Psychiatric Association, 2000). A number of attempts have been made to discover locomotor activity related markers of disorder.

For example, Walther et al. (2009) performed a quantitative activity analysis in patients with different schizophrenia subtypes, including paranoid, catatonic and disorganized. Activity was monitored for 24 h every 2 s, but only data during wakefulness were included in the main analysis. Three activity parameters were extracted; the mean number of activity counts per hour (AL), the percentage of epochs with non-zero activity (mobility index, MI), and the mean duration of immobility periods (MIP). Only schizophrenia subtype and type of anti-psychotic were found to have significant effect on AL, MI and MIP.

Hauge et al. (2011) analyzed differences in locomotor activity of schizophrenia patients, depressed patients and healthy subjects in two settings; (a) 1 min epochs recorded over 5 h, and (b) 1 h epochs recorded over 2 weeks. Sample Entropy and Fourier analysis were applied and have shown significantly different profiles of activity between all three groups.

Wulff et al. (2012) analyzed sleep and circadian rhythm disruption in schizophrenia outpatients in comparison to healthy unemployed individuals. Activity and light exposure were recorded every 2 min over 6 weeks. Activity data were analyzed using rest–activity characteristics, cosinor and periodogram analysis. Significant disruption in the sleep and rest–activity cycle was detected in all schizophrenia patients, independently of mood, mental state and anti-psychotic treatment.

While locomotor activity is a useful diagnostic criteria, another important factor of schizophrenia is disturbance of autonomic nervous system (ANS) as measured by cardiovascular variables, such as HR and heart rate variability (HRV). Existing research indicates that there is a relationship between cardiac activity and psychotic symptoms (Bär et al., 2008; Toichi et al., 1999, Valkonen-Korhonen et al., 2003).

Bär et al. (2008) studied the relationship between cardiovagal modulation and psychotic state in 40 unmedicated schizophrenia patients and 58 matched controls and found that the patients displaying stronger psychotic symptoms show increased HR, reduced HRV high frequency (HF) power and an increased LF/HF-ratio, which indicates an elevated sympathetic drive, an inhibited parasympathetic drive, or both. Such observations are consistent with stress as well as physiological illness.

Rachow et al. (2011) analyzed the interrelation of skin conductance levels and cardiac autonomic dysfunction with 18 unmedicated schizophrenia patients and 18 matched controls. They found significantly increased HR (consistent with results of Bär), decreased root mean square of the successive differences (RMSSD) in heart beats and decreased complexity (measured with compression entropy) of HR of schizophrenia patients in comparison to controls.

The fusion of locomotor activity and HR-related information has the potential to define a powerful model for schizophrenia, if they provide independent information. The study presented here attempted to identify the important features from these signals and evaluate the performance of a classifier for delineation between schizophrenia patients and control subjects. In doing so, it may be possible to develop a screening tool, help to identify severity of illness and track response to therapy and clinical status over time.

## Materials and methods

### Participants

Outpatients with diagnosis of schizophrenia (*N* = 16), all in relative symptomatic remission and on medication and healthy volunteers (*N* = 19) without a history of mental disorders (control group) were recruited for the study (Table 1).

**Table 1.** Demographic and clinical characteristics of study participants.

	Schizophrenia	Controls
Age (Mean ± SD)^b^	45.1 ± 12.3	51.7 ± 8.8
Gender^c^	58% male	75% male
Medication	All medicated^a^	N/A
Length of disease (years)	6.4 ± 3.4	N/A
Symptoms	All in relative symptomatic remission	No history of mental disorders

^a^Schizophrenia patients were prescribed anti-psychotic medication, including Olanzapine, Risperidone, Aripiprazole, Perphenazine, Fluphenazine, Ziprasidone, Haloperidol and Quetiapine. ^b^Difference between age distributions of schizophrenia and control groups is not significant (*p* = 0.09) using a two-sided Student *t-*test at a 95% confidence interval. ^c^Difference between gender distributions of schizophrenia and control groups is not significant (*p* = 0.31) using Fisher’s exact test.

**Figure 1.**
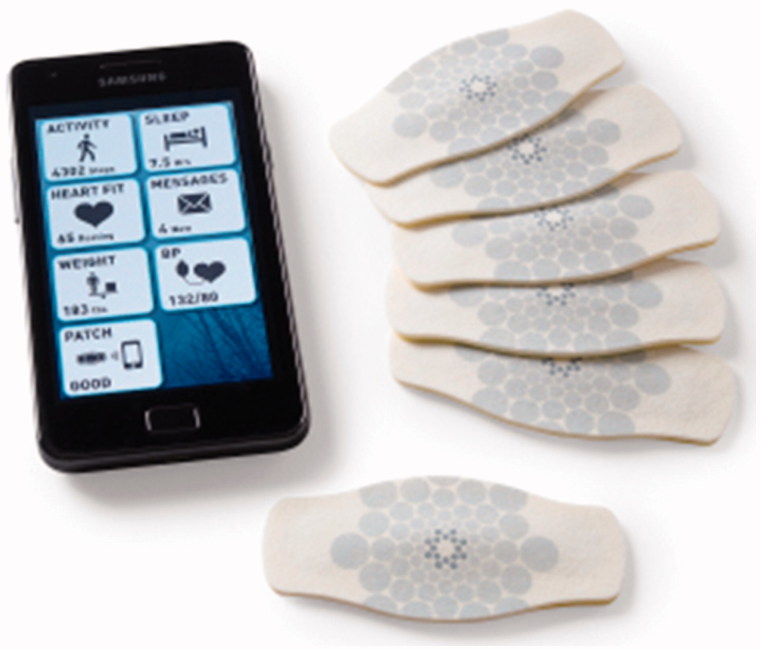
Adhesive activity and heart rate monitoring patch used in the study (Proteus Digital Health, Redwood City, CA).

Motor activity and HR of both groups were monitored for a maximum of 4 weeks using a disposable adhesive patch manufactured by Proteus Biomedical (Redwood City, CA) (Figure 1). The device was continuously worn on a body (chest) for at least 3 weeks and configured for data collection with the following settings:

- Electrocardiogram (ECG)-derived HR were collected approximately every 10 min by calculating mean HR on 15-s interval.
- Accelerometry-derived locomotor activity data were collected approximately every 5 min by calculating mean acceleration on 15-s interval.

All collected data were transmitted to a mobile phone via Bluetooth and further uploaded to a central server for processing. Data were collected in ambulatory settings and participants were engaging in their everyday activities, so in some cases measurements were affected by motion artifacts and discarded during the pre-processing stage.

### Data pre-processing

Locomotor activity data (recorded in arbitrary units, ranged from 0 to 1) and HR data (recorded in beats per minute, bpm) were collected with a variable recording rate and length. Since most analysis algorithms require evenly sampled data, the data were processed according to the following rules:

1. Data points with an interval, exceeding 1.5 × data collection rate (15 min for HR and 7.5 min for activity) were labelled as low quality and removed.
2. HR values lower than 20 bpm were labeled as low quality and removed, based on normative values of the human HR (Jensen-Urstad et al., 1997).
3. HR data were re-sampled to exactly 10-min intervals using zero-order hold (sample-and-hold) interpolation. Sample-and-hold interpolation approximates the next value in time series by replicating the previous value.
4. Locomotor activity data were re-sampled to exactly 5-min intervals using zero-order hold (sample-and-hold) interpolation.
5. For analysis, where synchronous activity and HR data were necessary, activity data were down-sampled to 10-min intervals using linear interpolation. Linear interpolation approximates the value in time series as a mean of previous and the next value.
6. The data from the device are influenced by motion artifacts, and high levels of activity are correlated to motion, including all data would reduce our ability to separate the independent influences of physiology and motion on the classifier performance. To avoid this bias, we performed an automated quality assessment on the HR data and removed subjects with more than 10% artifacts, consistent with Li et al. (2008). From complete recordings, the 10 d with the lowest percentage of missing data for that subject were selected. Recordings, where more than 10% of data were missing or were of low quality, were discarded.

### Data analysis

Analysis of activity data was performed in Matlab R2010a (Natick, MA). Signal processing algorithms were used to extract activity and HR features and classifiers were used to map these features into the schizophrenia and healthy controls groups. Four feature groups were selected, representing different dimensions of schizophrenia symptoms. The Wilcoxon rank sum test was used for testing significant differences between schizophrenics and normal controls and the Bonferroni multiple testing correction was applied to the results.

#### Statistical characteristics

Both locomotor activity and HR have been shown to be affected in schizophrenia (Bär et al., 2008; Hauge et al., 2011; Rachow et al., 2011; Walther et al., 2009), so statistical characteristics of HR and activity were calculated, including mean, median, mode, standard deviation (STD) and interquartile range (IQR).

#### Rest–activity characteristics

Recent studies indicate that circadian rhythm disruption plays a significant role in schizophrenia (Wulff et al., 2012), so the rest–activity characteristics of HR and activity, including levels during the Least Active 5 h (L5), Most Active 10 h (M10), Relative Amplitude (RA, Equation 1), Interday stability (IS, Equation 2) and Intraday variability (IV, Equation 3) (Witting et al., 1990; Van Someren, 1999) were included in the analysis.
(1) 
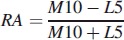

(2) 
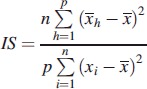

where *n* is the total number of data, *p* is the number of data per day, 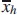 are hourly means, 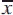 is the mean of all data, and *x_i_* represents the individual data points (Van Someren, 1999).
(3) 
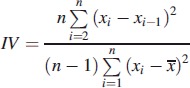

where definition of variables is the same as for Equation (2).

#### Multiscale entropy

Activity disorganization is one of the diagnostic criteria for schizophrenia (American Psychiatric Association, 2000). Hauge et al. (2011) indicated that entropy can be used to estimate such disorganization. Moreover, a study by Osipov et al. (2013) demonstrated that MSE applied to actigraphy provided information on disorganization. Therefore, MSE of activity and HR were included in the analysis.

MSE (Costa et al., 2005) applies sample entropy (*H_SE_*) (Richman & Moorman, 2000) calculation to a range of scales of original signal. *H_SE_* is an estimation of signal complexity, derived from the negative logarithm of the conditional probability of the appearance of longer patterns in a signal, considering presence of a shorter pattern. It is estimated by statistic
(4) 
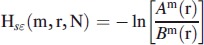

where *m* is the template length, *r* is the similarity threshold (or quantization level), *A^m^*(*r*) is a probability of matching (*m* + 1)-length template, *B^m^*(*r*) is a probability of matching *m*-length template and *N* is the length of the record. Two patterns of length *m* are considered as similar, if each point of a pattern in one part of the signal is within a distance *r* from the respective point in the other part of the signal.

Previous research indicates that the lower scales of MSE include the most information (Osipov et al., 2013), and hence the first five scales were calculated and coefficients of a third-degree polynomial fitted in a least squares sense into these scales, were used as features for further analysis. For an MSE analysis of biomedical signals, recommended values of the parameters are *m* = 1 or *m* = 2 and *r* being between 0.1 and 0.25 of STD (Aboy & Cuesta-Frau, 2007). In the previous study, it was identified that *m* = 1 and *r* = 0.1 of STD results in the best classification accuracy (Osipov et al., 2013), so these values were fixed accordingly.

#### Transfer entropy

To estimate the possible coupling between locomotor and cardiovascular activity, transfer entropy (TE) between HR and activity signals were calculated as follows
(5) 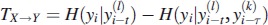

where *i* is a given point in time, *τ* and *t* are the time lags in *X* and *Y*, and *k* and *l* are the block lengths of past values in *X* and *Y*. TE estimates directional coupling between signals and measures the reduction of uncertainty, given past knowledge of a secondary signal in comparison with knowledge of a primary signal only. The algorithm is parameterized by primary and secondary signal lags (or the amount of history to take into account) (Schreiber, 2000).

Values of *k* and *l* were taken as *k* = 1 and *l* = 1 as recommended by Lee et al. (2012). The Darbellay–Vajda signal partitioning scheme (Darbellay & Vajda, 1999; Lee et al., 2012) was used for probability density function estimation. Coefficients of a third-degree polynomial, fitted in a least squares sense into the TE values with *t* = 1 and *τ* = {1,2,3,4,5}, were used as features for further analysis.

#### Feature selection and classification

Feature selection methods can be classified as filters and wrappers (Guyon & Elisseeff, 2003), where filters optimize some measure of feature usefulness and wrappers use the predictive model itself as a measure of feature selection quality. To maintain the principled manner of feature selection, in our study multivariate minimum-Redundancy-Maximum-Relevance (mRMR) criterion was used to identify the most important features for classification (Peng et al., 2005). This filter method allows selection of features that are predictive for the target class (maximum relevance), but at the same time they are orthogonal to each other (minimum redundancy), according to the Equations (6), (7), (8).
(6) 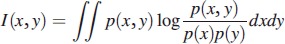

where *I(x;y)* is the mutual information between variables *x* and *y. p(x), p(y)* and *p(x,y)* are probability densities of these variables.

The relevance criterion is then given by:
(7) 
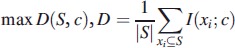

where *S* is a feature set with features *x_i_* and *c* is a target class (with *c* = 0 indicating normal, and *c* = 1 otherwise).

The redundancy criterion is then given by:
(8) 
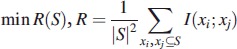

where *S* is a feature set with features *x_i_* and *x_j_*.

The relevance and redundancy criteria were combined using the Mutual Information Difference scheme and incremental search was then used to find features, which satisfy the above criteria. For the application of mRMR, features were discretized into five states between values of Mean ± STD, where STD is one of −1, −0.5, 0.5, 1 as suggested by Peng et al. (2005).

For classification into schizophrenia and normal controls categories, a support vector machine (SVM) with a Gaussian radial basis function (RBF) kernel was used (Cortes & Vapnik, 1995), *σ* = 4, selected based on previous work (Osipov et al., 2013). The SVM classifier attempts to create a hyperplane with a largest distance to nearest points in a feature space to separate target classes. If linear separation in the original feature space is not possible, features can be mapped into a higher dimensional space using kernel function, where separation is performed. Due to the limited number of samples in both schizophrenia and control classes, two-fold cross-validation with repeated random sub-sampling (Kohavi, 1995) was used to estimate the classification performance. Samples were randomly separated into the training and testing set and 1000 classification experiments performed to estimate the classification performance.

To evaluate the influence of combination of physiological and locomotor activity features, three feature selection and classification experiments were performed:

- Using HR features alone.
- Using locomotor activity features alone.
- Using HR, locomotor activity and transfer entropy features.

To evaluate the models, receiver operating characteristic (ROC) curves were created and the area under curve (AUC) was calculated for each model.

## Results

**Figure 2.**
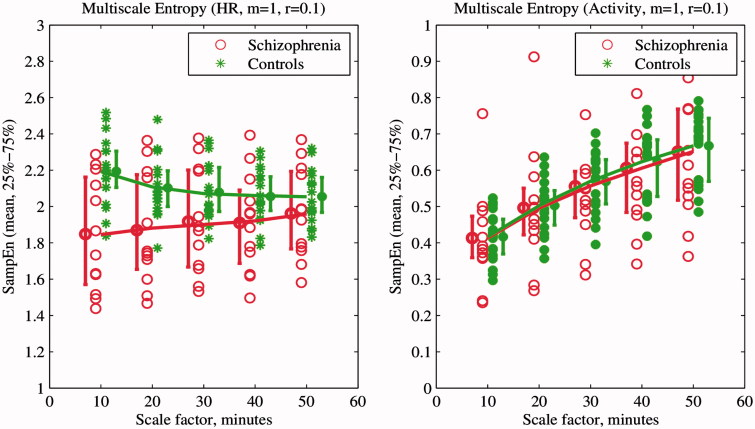
First five values of multiscale entropy of heart rate (left) and locomotor activity (right) signals of schizophrenia patients and normal controls. Solid lines represent fitting a third-degree polynomial in a least squares sense into the first five MSE scales, with coefficients presented in Table 2.

**Figure 3.**
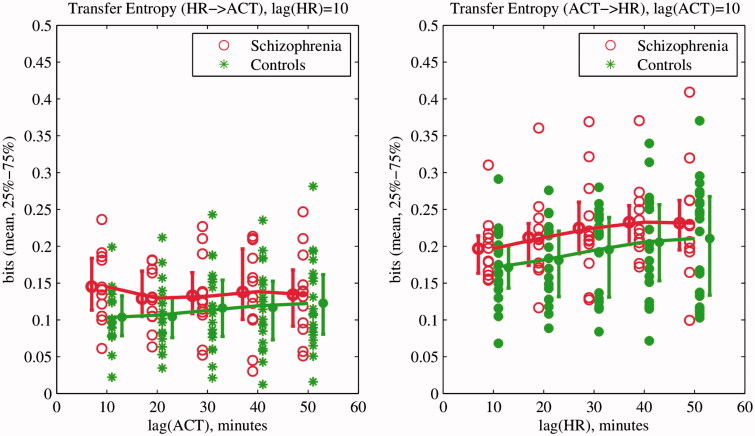
First five values of transfer entropy of heart rate (HR) and activity signals of schizophrenia patients and normal controls. Transfer Entropy from HR to locomotor activity is on the left and from locomotor activity to HR on the right. Solid lines represent the fitting of a third-degree polynomial in a least squares sense into the first five TE values. Resultant coefficients are presented in Table 2.

**Figure 4.**
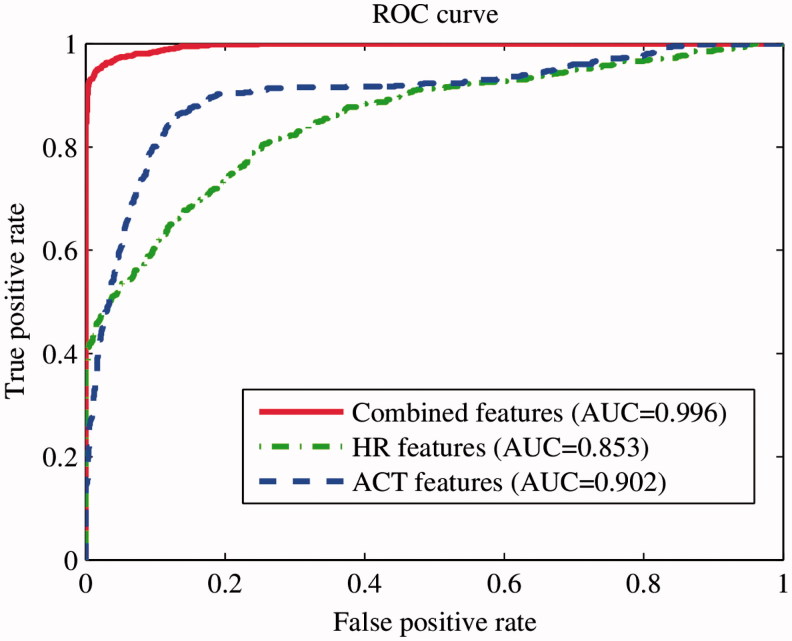
ROC curves and AUC for evaluated models based on locomotor activity only (ACT features; dashed line), heart rate only (HR features; dot-dashed line) and combined HR and activity features (solid line). The AUC values are significantly different between models at a 1% significance level according to two-sided Wilcoxon rank sum test with the Bonferroni correction for multiple comparisons.

After pre-processing, four records of schizophrenia subjects with an amount of missing data exceeding the 10% threshold were discarded. The missing data were probably caused by the poor contact of adhesive wearable sensor with patient’s skin and motion artifacts, and not related to the diagnosis of the patient. A total of 12 records of schizophrenia patients and 19 records of normal controls were processed for further analysis. Statistical characteristics as well as rest–activity characteristics of HR and locomotor activity signals were calculated as presented in Table 2. The results of the multiscale entropy and transfer entropy analysis together with feature selection and classification are presented in Table 2 and Figures 2 and 3. A ROC analysis performed with results is presented in Figure 4.

**Table 2.** Features of HR and locomotor activity of schizophrenia and control subjects (mean ± STD, arbitrary units for activity and bpm for HR) together with mRMR ranking^a^.

	Heart rate			Locomotor activity		
	Schizophrenia	Controls	Rank	Schizophrenia	Controls	Rank
Mean	82*.*01 ± 10*.*46	76*.*11 ± 6*.*19	4	0*.*04 ± 0*.*02	0*.*06 ± 0*.*01	15
Median	79*.*33 ± 10*.*83	74*.*70 ± 7*.*10	27	0*.*01 ± 0*.*00	0*.*02 ± 0*.*00	17
Mode	72*.*27 ± 12*.*71	69*.*23 ± 8*.*19	20	0*.*01 ± 0*.*00^c^	0*.*01 ± 0*.*00^c^	2
STD	13*.*95 ± 2*.*70	14*.*11 ± 2*.*23	21	0*.*08 ± 0*.*03	0*.*13 ± 0*.*03	**1**†
IQR	18.50 ± 5.00	19.71 ± 4.25	29	0.02 ± 0.02	0.03 ± 0.01	8
L5	1952.19 ± 249.99	1831.15 ± 162.50	12	1.17 ± 0.51	1.61 ± 0.33	9
M10	4311.61 ± 599.30	4016.32 ± 322.46	7	7.15 ± 2.71	10.05 ± 5.25	13
RA	0*.*38 ± 0*.*03	0*.*37 ± 0*.*02	18	0*.*71 ± 0*.*08	0*.*70 ± 0*.*06	33
IS	0*.*30 ± 0*.*14	0*.*36 ± 0*.*12	23	0*.*16 ± 0*.*04	0*.*18 ± 0*.*03	16
IV	0*.*60 ± 0*.*21	0*.*70 ± 0*.*23	19	1*.*23 ± 0*.*13	1*.*23 ± 0*.*23	28
MSE(1)^b^	<0*.*01	<0*.*01	5	<0*.*01	<0*.*01	31
MSE(2)^b^	−0*.*02 ± 0*.*12	0*.*05 ± 0*.*13	24	−0*.*02 ± 0*.*04	−0*.*01 ± 0*.*03	35
MSE(3)^b^	0*.*09 ± 0*.*34	−0*.*20 ± 0*.*36	14	0*.*13 ± 0*.*13	0*.*12 ± 0*.*09	26
MSE(4)^b^	1*.*78 ± 0*.*38	2*.*35 ± 0*.*34	**3**†	0*.*30 ± 0*.*11	0*.*31 ± 0*.*09	30
TE(1)^b^	<0*.*01	<0*.*01	10	<0.01	<0.01	32
TE(2)^b^	0*.*02 ± 0*.*04	0*.*00 ± 0*.*03	22	0*.*00 ± 0*.*06	0*.*01 ± 0*.*04	36
TE(3)^b^	−0*.*07 ± 0*.*11	−0*.*01 ± 0*.*07	6	0*.*01 ± 0*.*17	0*.*00 ± 0*.*13	25
TE(4)^b^	0*.*19 ± 0*.*15	0*.*11 ± 0*.*05	11	0*.*18 ± 0*.*11	0*.*17 ± 0*.*12	34

^a^Significantly different variables (*p* < 0.05) according to two-sided Wilcoxon rank sum test using the Bonferroni correction for multiple comparisons are marked with bold font and †. ^b^MSE(x) and TE(x) denote coefficients of a third-degree polynomial fitted in a least squares sense into MSE and TE values. ^c^Values are close to zero, but distributions of schizophrenia subjects and controls barely overlap, providing high feature ranking.

When using HR-derived features only, ordered by the mRMR algorithm, the two best features (including mean and MSE (4)) resulted in a 78.5% classification accuracy with 82.3% sensitivity and 72.2% specificity with an AUC of 0.85 (ROC curve presented in Figure 4). When using locomotor activity features only, ordered by the mRMR algorithm, the five best features, (STD, mode, IQR, IS and L5) resulted in a 85.5% classification accuracy with 85.3% sensitivity and 85.9% specificity with an AUC of 0.9 (ROC curve presented in Figure 4).

When HR, locomotor activity and transfer entropy features (ordered by the mRMR algorithm) were used to train an SVM, the four best features, (STD of locomotor activity, mode of locomotor activity, MSE (4) of HR and mean of HR) resulted in a 95.3% classification accuracy with 98% sensitivity and 91.1% specificity with an AUC of 0.99 (ROC curve presented in Figure 4).

## Discussion

Using a machine learning framework, the combination of HR and locomotor activity features provided the best classification accuracy, as can be seen from the Table 2, with almost a 10% increase in accuracy above using locomotor features alone (85.5% versus 95.3%). Our results are consistent with an earlier study, which we carried out on a similar patient population with actigraphy alone (Ospiov et al., 2013).

The optimal features set included different feature classes, representing levels of HR, locomotor activity and multiscale entropy dynamics across the time scales of HR. Consistent with previous results and theoretical expectations (Bär et al., 2008; Rachow et al., 2011), we found that HR is elevated in schizophrenia patients and the level of locomotor activity decreased. Despite the fact that HR elevation is not significant, it is an independent characteristic of schizophrenia group, as found by mRMR feature selection. The STD of locomotor activity was both significantly different and important from the point of view of classification, as well as the 4th coefficient of a polynomial fit of multiscale entropy for HR.

In contrast to previous results (Osipov et al., 2013), MSE of locomotor activity was not selected as a significant or a highly ranked feature, probably due to lower signal resolution (5-min sampling interval) or complimentary information embedded in the HR MSE. Similarly, transfer entropy differences were not important for classification with both activity and HR signals sampled every 10 min.

Since the number of individuals in our sample is relatively small compared to the number of features being used, we are unable to stratify by age and gender without loss of statistical power. Moreover, including age and gender in the model may have led to an artifactual relationship between patient classes and these variables. However, there is good reason to assume that neither age nor gender have an extremely significant impact on our results (age is 45.1 ± 12.3 for schizophrenia and 51.7 ± 8.8 for controls; 58% male in schizophrenia and 75% in control groups). HRV has been shown to be predominantly affected by mental activity (Bernardi et al., 2000; Clifford & Tarassenko, 2004), and the differences in age or gender in our study are small, so the gender imbalance should not be a significant factor in this work. MSE in particular, although known to exhibit a minor aging affect over one’s lifetime (Tobia et al., 2014), is not marked in the relatively small spread of ages between our control and patient groups. Therefore, we argue that the differences we observe are likely to be due to disease-related differences in activity and physiology, rather than any age- or gender-related phenomenon. We also note that there is no statistically significant difference in age and gender between the controls and the schizophrenic patients (Table 1).

It is interesting to note that the features presented here, when using probabilistic classifiers, or when converting the output into a probability, could perhaps be used as a metric of severity of disease, particularly for an individual, and therefore used to monitor response to therapy or to identify a psychotic relapse.

## Conclusions

As indicated by Teicher in his review of actigraphy and motion analysis use in psychiatry (Teicher, 1995), accurate monitoring of activity levels and circadian rhythms has a potential to aid clinicians in diagnosis; however, very few studies examined sensitivity and specificity of these features. Our research aimed to fill this gap and added another promising dimension by fusing the HR-based measures of ANS function with locomotor activity analysis and showed that physiological and physical activity signals provide complimentary information for classification of mental health.

However, more experimental data (from more individuals) and a higher sampling frequency (1 minute and higher) may be necessary to validate and improve these research findings. The study presented here also requires an independent validation where the diagnostic accuracy of the selected features will be estimated using the model with fixed parameters, found in this initial discovery set. Further research is also necessary on severity of particular symptoms, so that patients with different symptoms and symptom patterns can be analyzed separately.
